# Editorials

**Published:** 1891-09

**Authors:** 


### HOMŒOPATHIC PHYSICIAN,

A MONTHLY JOURNAL OF

HOMŒOPATHIC MATERIA MEDICA AND CLINICAL MEDICINE.

" If our school ever gives up the strict inductive method of Hahnemann, we are lost, and deserve only to be mentioned as a caricature in the history of medicine."—CONSTANTINE HERING.

Vol. XI. SEPTEMBER, 1891. No. 9.

### EDITORIALS.

"THE DOCTOR."—Allopathy has always claimed the high-sounding title, "scientific medicine." Let us endeavor to find out whether it is entitled to rank as a science, or as an art, or as empiricism.

"Science consists in an infallible and unchanging knowledge of phenomena."

"Art is a system formed from observation, and directed to a useful end."

"Empiricism is an unreasoning and instinctive imitation of previous practice."

Accepting the above as true, it requires no argument to show that allopathy is not science, for if there ever existed any system of therapeutics more changeable, and more given to drifting about in uncertainty, it has never yet been placed before the public. Its fallibility is shown by its own adherents in almost every clinical case reported by them, and when the question of therapeutics arises in any meeting in respect of any disease, there are as many opinions regarding what is best as there are speakers on the subject.

Hence, we need only accept their so-called science at their own valuation, in order to be able to determine that it is not "an infallible and unchanging knowledge of phenomena."

That it is an art, in that the conscientious allopaths think it is "directed to a useful end," we are willing to admit; but that the end is a large mortality table we are also forced to own.

That it is empiricism, in being "an unreasoning and instinctive imitation of previous practice," is recognized by the best writers of that school, even though they paradoxically use the term scientific in articles in which no great acuity of vision is necessary to see empiricism between all the lines.

We know of no allopathic writer who better represents the best thoughts of that school than Dr. Benjamin Ward Richardson, of London. Admiring him for his honesty of purpose, and his high attainments as an allopathic physician, we are, at the same time, from our Hahnemannian standpoint, obliged to declare that he is as far from scientific therapeutics as are his colleagues.

In a recent article Dr. Richardson describes a picture, which was on exhibition at the Royal Academy, entitled "The Doctor." Coming from the hand of Dr. Richardson we accept the description of "The Doctor" as applying to the allopathic doctor, and not to the doctor who applies in the treatment of the sick that law of nature first given to the world by Samuel Hahnemann, *similia similibus curantur.*

Dr. Richardson writes: "The central figure of the picture, the figure that makes and fills up the body of the picture, is 'The Doctor.' The name is happy, and by general acceptance and popular voice is correct; and yet, according to the strict meaning of the title, it is incorrect, for everything is done to show, not a doctor, in the original sense of a learned man, but an earnest, sympathetic, and thoughtful attendant on the sick. Every semblance of learning is put aside. There is no book, no philosophical instrument, no garment of learning. A common teacup and a bottle are all the instruments of aid that are in sight. The doctor himself is middle-aged, a strong, well-built, and a handsome man. He sits by the side of the sick couch, his eyes turned earnestly on his little patient, as if he were counting up the chances for life or for death, and as if the balance were as fine as it could be. He is a man too far in the valley of experience to be misled by enthusiasm, or to be led on by faith in what his skill can do; whilst at the same time he has seen so many strange recoveries when he least of all expected them, he is not as one without hope. If a layman were to ask him what is going to happen, he might reply, 'Well, there is youth on our side; and, prepared for the worst, we must act for the best;' but if the layman were clever enough to get at his actual mind, he would find him saying to himself, with the Danish Prince, 'Why, what an arrant knave and fool am I,' to sit here as a healer, powerless as the rest: or, thinking of other cases he has seen of the same nature, he may be trying to remember if any one plan of treatment has really been better than another. Evidently he hesitates, not as if he had done something and was waiting for the result, but as if he had not seen anything that could reasonably be done, and were waiting the action of that capricious jade, Nature, who, caring nothing for the woman's tears, nor the man's distraction, is pursuing her own relentless course."

That is the description of an allopathic doctor by a prominent allopathic doctor.

Could Dr. Richardson have said more plainly, Our art is but empiricism? Do we need more to show that allopathy can have no true idea of the "actual mind" of the doctor who is in possession of knowledge which enables him to know that he has done the best possible for his patient, the mind of him whose treatment of disease is based upon law, the Hahnemannian doctor?

Now look on this picture, the picture of the true doctor, the healer of the sick, who never "hesitates," but goes to the work in hand with a confidence begotten of experience in applying the only law of cure. With this confidence he approaches the bedside, knowing that if the patient be curable his proper application of the law will cure. There is but one thought in his mind, and that is, what is the remedy for this patient? His sympathies are, of course, with the patient; but he does not permit his sympathy to overcome his judgment, for he knows there is work before him that will require study. He listens to all that can be told him, the while closely observing the patient. He then notes, in writing, all that he hears and sees, and then begins his study for the remedy. He does not theorize regarding the pathology of the case, for he knows nothing is more misleading, but keeping constantly in view the one thing, what will cure? he presents a picture which in comparison to the above is in every respect superior. The farther he goes into "the valley of experience" the more enthusiastic he becomes regarding the help to be expected from the law of the similars, the more knowledge (not faith) he has of its helpfulness.

"If a layman were clever enough to get at his actual mind"—as he has reason for the faith that is in him, the layman may always "get at his actual mind"—he would *not* "find him saying to himself, 'Why, what an arrant knave and fool am I,' to sit here as a healer, powerless as the rest," for he knows that he is not powerless, for the more desperate the case the more closely he clings to his law, and thus his success in treating the sick is phenomenal. He never hesitates, for he has a guide, but he always sees something that can be done. He knows that "capricious jade, Nature," has laws, which, if given attention, will turn aside her caprice, and have her use all her powers to restore the sick. The first of these laws is to avoid crude drugs. Thus, by not overwhelming that "capricious jade" by vile nostrums, there will be no cause for the "woman's tears and the man's distraction," hence in the picture of the true doctor tears and distraction will not appear, but instead there will be pictured in the faces of all the joy and confidence which comes from doing the right, the best that can be done, which is done by following the teachings of Hahnemann. G. H. C.

---

To Correspondents.—We must ask the indulgence of all who have favored us with their correspondence, for our seeming neglect to answer their communications. We have been lately confined to bed by sickness from over-work, and have been obliged to leave our office duties for a short season of rest and recuperation. We can assure them that every letter will surely be answered, though the answer may be long delayed. No communication coming to this office will be ignored. W. M. J.

